# Insight into the Secondary Metabolites of *Geum urbanum* L. and *Geum rivale* L. Seeds (Rosaceae)

**DOI:** 10.3390/plants10061219

**Published:** 2021-06-15

**Authors:** Marek Bunse, Peter Lorenz, Florian C. Stintzing, Dietmar R. Kammerer

**Affiliations:** 1 Department of Analytical Development & Research, Section Phytochemical Research, WALA Heilmittel GmbH, Dorfstr. 1, DE-73087 Bad Boll/Eckwälden, Germany; Marek.Bunse@wala.de (M.B.); Peter.Lorenz@wala.de (P.L.); Florian.Stintzing@wala.de (F.C.S.); 2 Department of Plant Systems Biology, Hohenheim University, Garbenstraße 30, DE-70599 Stuttgart, Germany

**Keywords:** avens, seeds, specialty oils, phenolics, triterpenes, asiatic acid, fatty acids, γ-linolenic acid, RP-HPLC-DAD/ESI-MS^n^, GC/MS

## Abstract

The present study aimed at the identification and quantitation of phenolic compounds, fatty acids, and further characteristic substances in the seeds of *Geum urbanum* L. and *Geum rivale* L. For this purpose, individual components of extracts recovered with MeOH, CH_2_Cl_2_, and by cold-pressing, respectively, were characterized by HPLC-DAD/ESI-MS^n^ and GC/MS and compared with reference compounds. For both *Geum* species, phenolic compounds, such as flavonoids and gallic acid derivatives, and triterpenes, such as saponins and their aglycones, were detected. Surprisingly, both *Geum* species revealed the presence of derivatives of the triterpenoid aglycons asiatic acid and madecassic acid, which were characterized for the first time in the genus *Geum*. Furthermore, the fatty acids of both species were characterized by GC–MS after derivatization. Both species showed a promising fatty-acid profile in terms of nutritional properties because of high proportions of unsaturated fatty acids. Linoleic acid and linolenic acid were most abundant, among other compounds such as palmitic acid and stearic acid. In summary, the present study demonstrates the seeds of *G. urbanum* and *G. rivale* to be a valuable source of unsaturated fatty acids and bioactive phenolics, which might be exploited for nutritional and cosmetic products and for phytotherapeutic purposes.

## 1. Introduction

*Geum* L., commonly called avens, is a genus in the Rosaceae family, subfamily Rosoideae, which comprises about 55 species of rhizome forming perennial herbaceous plants. The genus *Geum* is widespread across Eurasia, North and South America, and Africa. Avens species are found in exposed vegetation and forests and sometimes also as “weeds”. The species *G. montanum* L. (alpine avens), *G. reptans* L. (creeping avens), *G. urbanum* L. (wood avens), and *G. rivale* L. (water avens) are found as part of the Central European flora. *G. urbanum* and *G. rivale* are among the best-known European species, which are also used in pharmaceutical applications, especially in complementary medicine such as phytotherapy. For this purpose, mainly the roots and rhizomes (in the following text summarized as roots), but also aerial parts of the two species are processed. *G. urbanum* and *G. rivale* have long been used in traditional European medicine for the treatment of diarrhea, stomach complaints, febrile diseases, gingivitis, and inflammation of mucous membranes [1]. In addition, the roots of wood avens and water avens have been used as a substitute for clove because of their eugenol content, as an additive to spirits and liqueurs in the food industry, and as cosmetic ingredients of toothpastes and mouthwashes [1]. The main secondary metabolites of the roots and herbal parts reported so far are gallo- and ellagitannins, procyanidins and other polyphenolics, ascorbic acid, and essential oil components, such as eugenol [2]. The high tannin content is typical for the Rosaceae family. More recent studies, especially on *G. urbanum*, have reported anti-inflammatory, antimicrobial, antioxidant, neuroprotective, and hypotensive effects [2]. It is, therefore, surprising that the phenolic compound and fatty-acid profiles of the seeds of these two species have neither been studied nor used for pharmaceutical, nutritional, or culinary purposes. Therefore, this study aimed at a profound characterization of the secondary metabolite profile of the seeds of *G. urbanum* and *G. rivale*, focusing particularly on phenolic compounds, such as monophenol structures and ellagitannins, as well as triterpenes and their derivatives. Furthermore, the fatty-acid profile was analyzed and compared between the two *Geum* species. In addition, two extraction methods, i.e., solvent extraction and cold-pressing, should be assessed with regard to seed oil yields and their compound profiles. Although *Geum* is known to be rich in phenolics, and extracts derived therefrom exert various pharmaceutical activities to treat several diseases, there is still a lack of comprehensive knowledge concerning the phytochemical composition of the seeds. The latter are expected to be potential sources of secondary metabolites, thus rendering them promising candidates for applications in the food, cosmetic, and pharmaceutical sectors.

## 2. Results and Discussion

### 2.1. GC/MS Analyses of Fatty Acids

**Figure 1:**
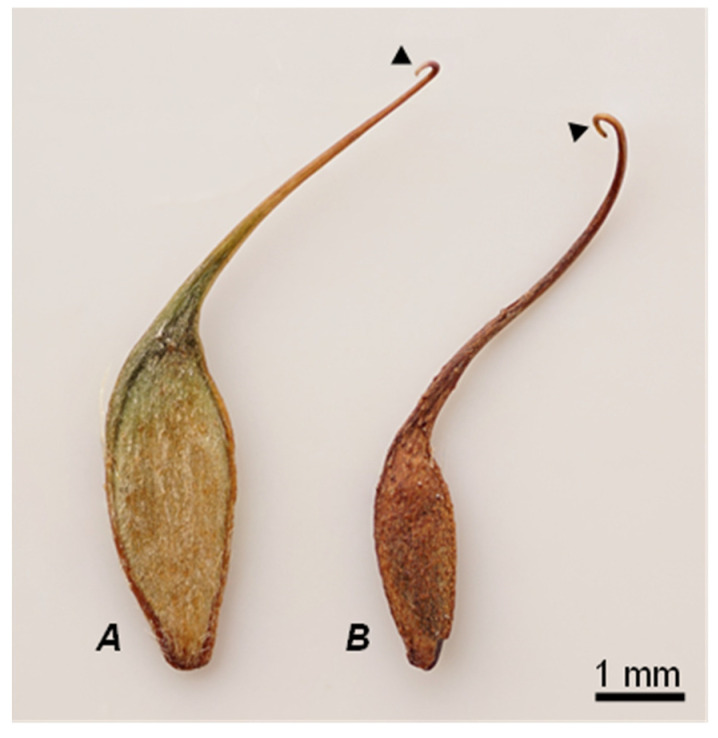
Seeds of *Geum urbanum* L. (**A**) and *Geum rivale* L. (**B**) with their typical morphologic adaptations (spikes, hooks, or barbed projections (▶). The scale bar shown corresponds to 1 mm.

The diaspores (fruit) of *G. urbanum* and *G. rivale* (Figure 1) are randomly spread by animals. For this reason, they have some morphologic adaptations such as spikes, hooks, barbed projections, or awns [3]. Furthermore, seeds store proteins, carbohydrates, phosphates, and lipids that act as the carbon skeleton and energy source, e.g., for germination [4]. To analyze the fatty-acid compositions, seeds were defatted with CH_2_Cl_2_ or cold-pressed, and the oils obtained were analyzed by chromatographic and spectrometric methods. 

**Figure 2:**
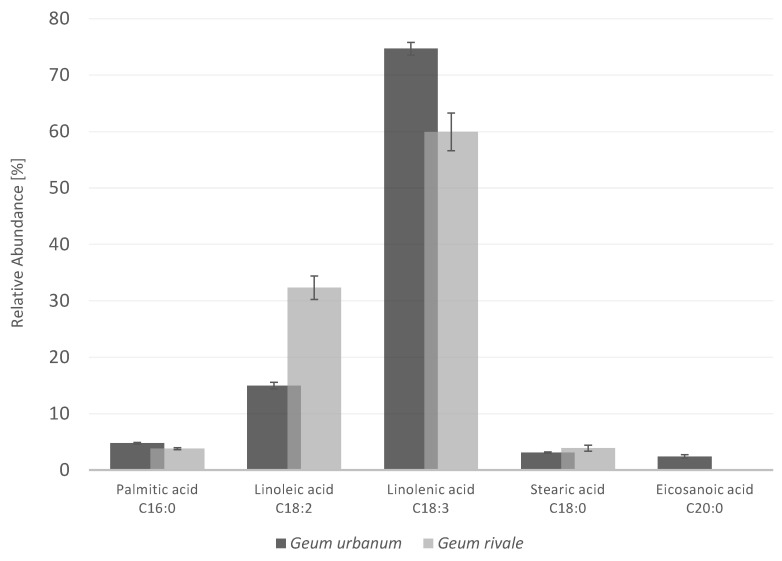
Relative abundance (%) of fatty acids in the seeds of *Geum urbanum* L. and *Geum rivale* L. (CH_2_Cl_2_ extracted seed oils). Standard errors are given (*n* = 3).

The fatty-acid composition of the two species revealed the presence of palmitic acid, linoleic acid, α-linolenic acid, and stearic acid as major components, which were assigned on the basis of their mass spectra and a comparison with those of reference compounds and with the NIST database. The relative proportions of individual fatty acids (Figure 2) of the two species were almost identical for palmitic acid (*G. urbanum*: 5%; *G. rivale*: 4%) and stearic acid (*G. urbanum*: 3%; *G. rivale*: 4%). In contrast, the proportions of linoleic acid (*G. urbanum*: 15%; *G. rivale*: 32%), α-linolenic acid (*G. urbanum*: 75%; *G. rivale*: 60%), and eicosanoic acid (*G. urbanum*: 2%; *G. rivale*: 0%) varied considerably. 

The two *Geum* species differed in their relative amounts of C18:2, which was twice as high in *G. rivale* as compared to *G. urbanum*, whereas the former was devoid of C20:0. Furthermore, the two cold-pressed oils of *G. urbanum* and *G. rivale* seeds were compared in the same way (Figure 2). The fatty-acid profile of the cold-pressed oils was almost identical to that of the oil samples resulting from CH_2_Cl_2_ extraction. The cold-pressed oil of *G. urbanum* revealed proportions of 4% palmitic acid, 16% linoleic acid, 77% linolenic acid, 2% stearic acid, and <1% eicosanoic acid, whereas the proportions for *G. rivale* were as follows: 5% palmitic acid, 47% linoleic acid, 45% linolenic acid, 3% stearic acid, and <1% eicosanoic acid. Interestingly, neither *γ*-linolenic acid (GLA; C18:3) nor oleic acid (C18:1) was detected in any of the samples according to a comparison with reference compounds. The high abundance of unsaturated fatty acids indicates a high-quality oil from a nutritional viewpoint. Furthermore, the cold-pressed oil of *G. urbanum* with its dark green color and high viscosity differed markedly from the oil obtained upon solvent extraction, which showed much lower viscosity. The latter parameter is significantly affected by the fatty-acid profile. Among other things, fatty acids can form crystalline structures, which have a marked impact on oil viscosity [5].

### 2.2. HPLC-DAD/MS^n^ Analysis of Phenolic Compounds and Triterpenoids

**Figure 3:**
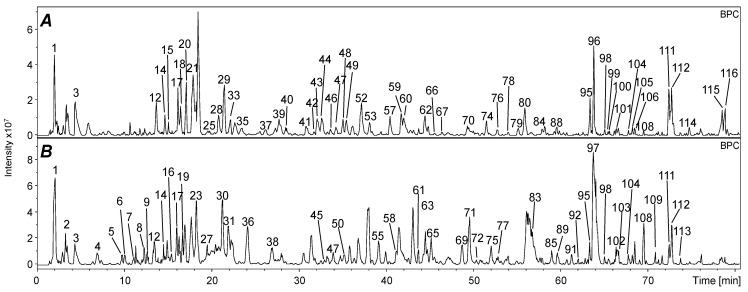
LC/MS^n^ chromatograms (BPC) of phenolic compounds in MeOH seed extracts of *Geum urbanum* (**A**) and *Geum rivale* (**B**). For compound assignment, see Table 1. For the sake of clarity, not all assigned compounds are numbered. According to the retention times shown in Table 1, the corresponding peaks could be assigned.

In this study, MeOH extracts of *G. urbanum* and *G. rivale* seeds were studied in detail regarding their phenolic compound profile (Figure 3). For this purpose, the seeds were defatted (CH_2_Cl_2_) and subsequently extracted with MeOH. Then, the methanolic extracts were subjected to analysis by HPLC-DAD/MS^n^ to characterize individual phenolic compounds. Furthermore, the cold-pressed oils of both *Geum* species were extracted with MeOH and compared by LC/MS^n^. In summary, more than 100 individual compounds were characterized and tentatively assigned to these fractions on the basis of their retention times (*t_R_*), UV/Vis spectra, mass-to-charge ratios (negative ionization mode), and their specific fragmentation patterns in comparison with bibliographic references (Table 1).

**Figure 4:**
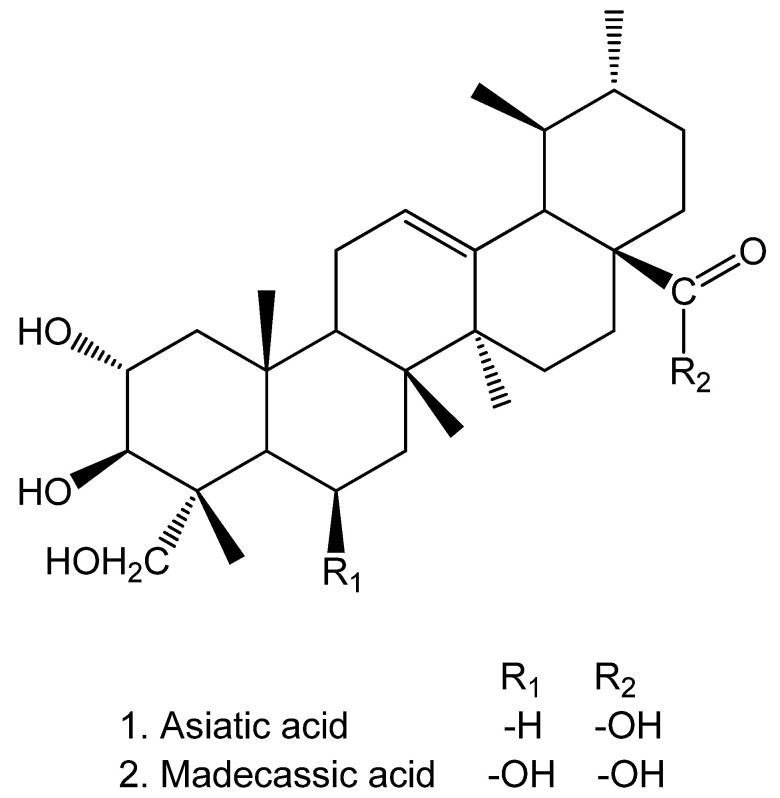
Molecular structures of asiatic acid and madecassic acid.

The tentatively assigned compounds of the MeOH extracts of both species belonged to various classes of phenolics including hydroxybenzoic acid and hydroxycinnamic acid derivatives, flavonoids, and ellagitannins. Most of the detected phenolic constituents were characteristic of Rosaceae species rich in tannins. In plants, the biological function of these polyphenolics is mostly based on their protective capabilities against herbivores, pathogens, and UV-B radiation [6]. The main constituents are based on gallic acid core structures (Table 1), which are well known for the genus *Geum*. Moreover, some triterpenoids and their derivatives (*t_R_* = 61.9–78.6 min) could be tentatively assigned in *Geum* for the first time. The core structures of these triterpenoids belong to asiatic acid (AA) and madecassic acid (MA; Figure 4). The fragmentation patterns of 26 derivatives of AA and MA were compared with the reference standard of MA and literature data. Asiatic acid and its derivatives were first mentioned as secondary metabolites of *Centella asiatica*, an herbaceous, frost-susceptible perennial plant in the Apiaceae family [7]. AA is the most prominent constituent of *Centella* and possesses biological activities, notably anticancer, anti-inflammatory, wound healing, antidiabetic, antioxidant, hepatoprotective, anti-hepatitis C virus, and neuroprotective properties [7]. The triterpenoid derivatives of AA and MA were characterized in *Geum* for the first time. However, it was not possible to clearly assign all individual derivatives. The MA reference standard showed a peak cluster in a retention time range of *t_R_* = 67.8–70.0 min revealing fragment ions at *m*/*z* 503, 437, 407, 392, 363, and 159. In previous studies, 19 metabolites of AA and MA could be identified in addition to AA and MA with similar fragmentation patterns [8]. These were mainly formed by hydroxylation, dehydrogenation, dihydroxylation, and combinations of these reactions as a result of the metabolic capability of zebrafish (feeding study) [8].

**Table 1:** Peak assignment of metabolites detected in MeOH extracts of *Geum urbanum* (A: methanolic extract of defatted seeds; A*: methanolic extract of cold-pressed seed oil) and *Geum rivale* (B: methanolic extract of defatted seeds; B*: methanolic extract of cold-pressed seed oil) using HPLC-DAD/ESI-MS^n^ (negative ionization mode).

Peak No. ^a^	Compound ^b^	*t_R_* (min)	MS (*m*/*z*)	MS/MS (*m*/*z*)	A	B	A*	B*	Reference
**Sugars**									
1	hexose polymer	2.1	683	683, 533, 445, 377, 341, 179, 161, 131, 113, 101, 89, 59	✓	✓			[9]
33	saccharide	22.2	431	431, 387, 287, 225, 179, 161, 143, 131, 113, 101, 89, 59	✓				[10]
56	saccharide	40.1	547	547, 311, 293, 221, 191, 147, 131, 101, 89		✓		✓	[11]
**Phenolics**									
2	HHDP ^c^-*O*-hexoside	3.6	481	481, 421, 301, 284, 257, 229, 201, 185		✓			[12]
4	galloyl-hexoside	7.1	331	331, 313, 271, 211, 193, 169, 125		✓			[13]
5	galloyl-hexoside	9.8	331	331, 301, 169, 125		✓			[13]
6	galloyl-HHDP-hexoside	10.2	633	633, 481, 301, 184, 257, 229, 201, 185		✓			[14]
7	digalloyl-hexoside	11.1	483	483, 429, 331, 313, 271, 241, 211, 193, 169, 125		✓			[13]
8	3,4-dihydroxybenzoic acid-*O*-hexoside	12.3	315	315, 279, 225, 153, 109, 108		✓			[15]
9	galloyl-HHDP-hexoside	12.6	633	633, 483, 435, 397, 345, 301, 284, 257, 229, 185, 137		✓			[14]
10	galloyl-HHDP-hexoside	12.7	633	633, 481, 436, 301, 257, 229, 185, 123		✓			[14]
11	galloyl-hexoside	12.8	331	331, 285, 169, 153, 125		✓			[14]
12	pedunculagin	13.7	783	783, 481, 301 257, 229, 185, 157	✓	✓			[16]
13	3,4-dihydroxybenzoic acid-*O*-hexoside	13.9	315	315, 271, 203, 153, 109		✓			[15]
14	galloyl-HHDP-glucose (corilagin isomer)	14.7	633	633, 481, 301, 284, 275, 257, 229, 185, 159	✓	✓			[16]
15	7-methoxy-3′, 4′-dihydroxyl flavanone	15.0	285	285, 153, 109	✓				[17]
16	galloyl-HHDP-hexoside	15.4	633	633, 481, 301, 275, 273, 229, 201,185		✓			[14]
17	galloyl-HHDP-hexoside	16.2	633	633, 391, 301, 275, 273, 257, 229, 201, 185	✓	✓			[14,16]
18	pedunculagin	16.5	783	783, 481, 301, 275, 257, 229, 185	✓	✓			[16]
19	oxyresveratrol-*O*-hexoside	16.7	451	451, 405, 327, 243, 225, 179, 167, 149, 134, 113		✓			[18]
20	procyanidin B1/B2	17.0	577	577, 451, 425, 407, 289, 257, 241, 213	✓	✓			[16,19,20,21]
21	procyanidin B1/B2	17.9	577	577, 451, 425, 407, 289, 285, 257, 213	✓	✓			[16,19,20,21]
22	apigenin pentoside	18.3	447	447, 401 [M − H] − 46, 287, 161, 131, 113	✓				[22]
23	epi-/catechin	18.3	289	289, 245, 227, 205, 203, 187, 161, 123		✓	✓	✓	[21]
24	digalloyl-HHDP-hexoside	18.4	483	183, 392, 313, 289, 271, 211, 169, 168, 124		✓			[14,23]
25	catechin	18.5	289	289, 271, 245, 231, 227, 203, 188, 161	✓				[24]
26	digalloyl-HHDP-hexoside	18.6	785	785, 483, 419, 331, 301, 284, 257, 229, 186, 158		✓			[23]
	5-caffeoylquinic acid	18.9	353	353, 191, 179, 173, 127				✓	[25]
27	luteolin-hexoside	19.6	447	447, 401, 285, 269, 233, 161, 101		✓			[26]
28	galloyl-HHDP-hexoside	20.8	633	633, 481, 463, 301, 283, 257, 229, 201, 185, 162	✓	✓			[16,23]
30	luteolin-hexoside	21.5	447	447, 431, 361, 285, 257, 241, 217, 213, 163, 109		✓			[26]
35	(epi)afzelechin-(epi)catechin	23.5	561	561, 543, 491, 429, 435, 425, 407, 381, 329, 289, 271, 245, 227, 203, 187, 179, 125	✓				[21]
	naringenin	23.9	271	271, 269, 225, 151, 85				✓	[27]
36	cyanidin 3-*O*-hexoside	24.3	465	465 [−18], 339, 303, 285, 241, 213, 199, 169		✓			[28]
	apigenin	26.1	269	269, 225, 207, 151				✓	[29]
38	digalloyl-HHDP-hexoside	27.1	785	785, 633, 483, 419, 301, 257, 229, 185		✓			[23]
39	HHDP-hexoside	27.7	482	482, 461, 444, 368, 301, 275, 257, 229, 203, 175, 169	✓				[13]
40	casuarinin or casuariin	28.6	612	612, 603, 573, 527, 458, 301, 275, 257, 229, 211, 169	✓				[14]
41	trigalloyl-HHDP-hexoside	30.9	937	784, 937, 767, 741, 613, 589, 465, 301, 275	✓	✓			[14]
42	galloyl-HHDP-hexoside	31.7	635	635, 618, 465, 313, 295, 235, 193, 169, 125	✓	✓			[23]
	naringin	31.9	581	581, 563, 545, 515, 445, 401, 383, 357, 321 265, 223, 195 179				✓	[30]
43	ellagic acid hexuronide	32.2	477	477, 301, 284, 257, 229, 201, 185, 174	✓		✓		[23]
44	ellagic acid hexoside	32.7	463	463, 301, 284, 257, 229, 201, 185, 173, 145	✓	✓	✓		[13]
45	catechin-*O*-galloyl dimer	33.0	729	729, 635, 577, 559, 451, 425, 407, 363, 285		✓			[15]
46	casuarinin or casuariin	33.7	612 (935)	612, 603, 573, 555, 527, 458, 437, 379, 301, 275, 257, 229, 185, 157	✓	✓			[14]
47	ellagitannin (tentatively assigned)	34.3	552	552, 530, 468, 392, 316, 301, 169	✓	✓			
48	quercetin/ellagic acid-*O*-(*O*-galloyl)-hexoside	35.0	615	615, 463, 392, 301, 257, 229, 185	✓	✓			[31]
49	catechin/epicatechin dimer	35.4	577	577, 551, 451, 425, 407, 381, 363, 297, 285, 281, 255, 213	✓				[15,32]
50	galloyl-bis-HHDP-hexoside	35.3	935	935, 633, 551, 435, 301, 284, 229		✓			[14]
51	galloyl-bis-HHDP-hexoside	36.1	935/784	784, 935, 633, 465, 421, 313, 301, 252, 221, 169, 137	✓				[33]
52	HHDP-/ellagic acid derivative	37.2	935/467	467, 441, 391, 301, 275, 271, 257, 227, 169, 125	✓				[23]
54	trigalloyl-HHDP-hexoside	38.4	937/784	784, 937, 613, 557, 461, 417, 399, 227, 200, 171	✓				[23]
55	sinapic acid derivative	39.3	403	403, 223, 205, 179, 161, 135		✓		✓	[34]
57	ellagic acid pentoside	40.5	433	433, 301, 284, 273, 257, 244, 229, 201, 185, 201, 185, 173	✓		✓		[16]
58	isorhamnetin-*O*-hexoside	41.3	477	477, 315, 300, 272, 244		✓			[23]
59	digalloyl-HHDP-hexoside	41.7	767	767, 615, 467, 465, 767, 465, 392, 301, 169	✓	✓			[23]
60	ellagic acid	42.1	301	301, 284, 257, 229, 201, 185	✓		✓		[16]
	undefined ellagitannin	43.7	467	467,.458, 382, 301, 275, 257, 229, 169			✓	✓	[13]
61	caffeoylquinate shikimate derivative	43.8	509	509, 491, 473, 367, 339, 313, 167, 149	✓				[35]
62	quercetin glucoside/rhamnoside	44.5	467	467, 458, 382, 319, 301, 284, 275, 257, 229, 201, 185, 151	✓	✓	✓		[36]
63	quercetin-hexoside	44.6	463	463, 303, 301, 271, 255, 229, 179, 151, 107		✓		✓	[27]
	quercetin-*O*-glucuronide	44.6	477	477, 301, 273, 257, 229, 211, 193, 179, 151				✓	[37]
64	trigalloyl hexose	44.9	617	393, 617, 465,449, 317, 313, 246, 169	✓	✓			[23]
65	caffeoylglucaric acid	45.3	417	417, 371, 209, 179, 161, 159, 113		✓		✓	[38]
67	epicatechin-3-*O*-gallate	46.4	441	441, 317, 289, 245, 205, 203, 188, 179, 137	✓				[19]
69	isorhamnetin pentoside	48.8	447	447, 315, 300, 272, 244, 228, 200, 185		✓			[27]
70	ellagic acid derivative	49.3	489	489, 476, 439, 301, 284, 257, 229, 185	✓		✓		
	kaempferol-*O*-hexoside	49.4	447	447, 385, 327, 285, 255, 227, 213, 193, 173, 151				✓	[39]
71	tetragalloyl-hexoside	49.7	769	469, 769, 617, 465, 317, 295, 241, 169		✓			[23]
72	isorhamnetin pentoside	50.4	447	447, 381, 315, 300		✓			[27]
73	monogalloyl-hexoside derivative	50.9	521	521, 469, 331, 271, 211, 168, 124		✓			[23]
74	ellagic acid derivative	51.5	489	489, 467, 439, 301, 300, 184, 271, 257, 244, 229, 229, 201, 185, 160	✓		✓		
	methylquercetin-*O*-hexuronide	51.5	491	491, 315, 300, 255, 175				✓	[29]
75	diosmetin-7-*O*-hexoside	52.2	461	461, 445, 377, 328, 313, 298		✓		✓	[40]
77	*p*-coumaroylshikimic acid derivative (tentatively assigned)	53.0	527	527, 503, 469, 423, 361, 319, 301, 273, 271, 256, 215		✓			
78	kaempferol-3-*O*-hexoside	54.0	591	591, 571, 553, 529, 489, 447, 285, 257, 229, 197, 163	✓	✓	✓	✓	[41]
83	dimethylellagic acid-sulfate	57.0	409	409, 329, 314, 299, 271		✓		✓	[27]
84	methylellagic acid pentoside derivative (tentatively assigned)	58.2	503	503, 443, 435, 315, 300, 271, 244	✓		✓		[42]
85	HHDP-hexoside derivative	59.1	452	452, 376, 316, 301, 275, 249, 183, 169, 125		✓			[13]
86	catechin derivative	59.3	333	333, 315, 289, 288, 259, 245, 233, 231, 217, 200, 173	✓				
88	quercetin-derivative	59.9	542	542, 521, 457, 405, 319, 301, 284, 271, 257, 229, 201, 185, 129	✓				[43]
	quercetin	60.5	301	301, 273, 229, 213, 193, 151, 121			✓		
	quinic acid derivative	61.2	253	253, 235, 209, 191, 135, 93			✓		
91	rhamnazin	61.4	329	329, 314, 299, 271		✓		✓	[44]
	flavonoid	61.7	271	271, 253, 227, 185			✓		
93	kaempferol deoxyhexosylhexoside	62.1	593	593, 447, 285, 257, 182, 151		✓	✓		[45]
94	undefined ellagitannin derivative	62.9	444	444, 397, 368, 301, 275, 229, 213, 169, 121		✓			
	naringenin	63.3	271	271, 177, 151, 107			✓	✓	[15]
97	5,6-dihydroxy-3′,4′,7-trimethoxyflavone sulfate	63.9	423	423, 343, 328, 313		✓			[43]
98	3′,5′-*O*-dimethyltricetin	65.0	329	329, 311, 293, 229, 211, 183, 171, 155, 127	✓	✓	✓	✓	[46]
	quercetin isomer	65.8	301	301, 283, 265, 257, 239, 221, 187, 151, 127, 125, 113, 97			✓	✓	
	flavonoid derivative	66.2	287	287, 269, 241, 221, 211, 139, 125, 9, 97, 85			✓	✓	
102	5,6-dihydroxy-3′,4′,7-trimethoxyflavone	66.5	343	343, 328, 313, 298, 257		✓			[43]
**Lignan**									
31 ^d^	(+)-pinoresinol-*O*-hexoside, (+)-epipinoresinol-4′’-*O*-hexoside and (+)-epipinoresinol-4′-*O*-hexoside	22.0	565	565, 519, 387, 251, 225, 179, 161, 113		✓		✓	[40]
**Terpenes**									
32	geniposide	22.1	433	433, 387, 225, 207, 189, 179, 153, 125		✓			[47]
53	8′-hydroxy-abscisic acid hexoside	38.1	441	441, 397, 365, 330, 205, 179, 161, 150, 139, 113, 101	✓	✓	✓	✓	[48]
80	triterpene acid-*O*-hexoside acetyl	55.9	711	711, 665 [M − H] − 46, 503, 485, 453, 441, 409, 407, 379, 363, 333	✓		✓	✓	[49]
81	ganoderic acid C2 hexoside	56.5	679	679, 633, 591, 573, 551, 517, 499, 481, 455, 441, 397, 381, 365, 297	✓				[12]
89	triterpene acid-*O*-hexoside	59.9	709	709, 663 [M − H] − 46, 501, 457, 425, 409, 395, 353, 341, 229, 149		✓			[12]
90	triterpene acid-*O*-hexoside	60.8	711	711, 665 [M − H] − 46, 503, 457, 441, 421, 403, 375		✓			[12]
92	asiatic acid/madecassic acid derivative	61.9	709	709, 663 [M − H] − 46, 501, 457, 427, 409, 391, 379, 363, 347		✓			[12]
95	asiatic acid/madecassic acid derivative	63.4	695	695, 649 [M − H] − 46, 487, 469, 441, 423, 405, 393, 377	✓	✓			[8]
96	asiatic acid/madecassic acid derivative	63.8	695	695, 649, 487, 469, 437, 423, 405, 393; 369	✓		✓		[8]
	asiatic acid/madecassic acid derivative	64.6	503	503, 485, 459, 441, 423, 405, 389, 369, 351, 321			✓		
99	asiatic acid/madecassic acid derivative	65.4	695	695, 559, 487, 441, 423, 377, 153	✓				[8]
100	asiatic acid/madecassic acid derivative	65.5	693	693, 647, 643, 559, 503, 485, 441, 409, 392, 367, 325, 266	✓				[8]
	asiatic acid/madecassic acid derivative	65.7	503	503, 485, 439, 423, 407, 397, 383, 369, 351, 339, 285			✓		
101	asiatic acid/madecassic acid derivative	66.4	503	503, 485, 453, 439, 421, 409, 355	✓		✓	✓	[8]
103	asiatic acid/madecassic acid derivative	66.7	693	693, 647, 503, 485, 467, 439, 423, 393, 365	✓	✓			[8]
104	asiatic acid/madecassic acid derivative	67.8	503	503, 485, 459, 441, 421, 403, 393, 359, 307, 291, 145	✓	✓	✓	✓	[8]
105	asiatic acid/madecassic acid derivative	68.3	501	501, 483, 471, 453, 439, 421, 405, 403, 365, 229	✓	✓	✓	✓	[8]
106	asiatic acid/madecassic acid derivative	68.6	503	503, 485, 441, 421, 409, 403, 393, 378, 375, 317, 268	✓	✓	✓	✓	[8]
	asiatic acid/madecassic acid derivative	68.8	501	501, 483, 471, 439, 421, 409, 378, 355			✓	✓	
107	asiatic acid/madecassic acid derivative	69.1	457	457, 437, 409, 393, 365, 323, 321, 163, 149		✓			[8]
	asiatic acid/madecassic acid derivative	69.1	489	489, 471, 469, 445, 429, 427, 425, 395, 369, 355,325			✓		
108	asiatic acid/madecassic acid derivative	69.5	503	503, 485, 465, 437, 421, 419, 402, 391, 176, 361	✓	✓	✓	✓	[8]
	asiatic acid/madecassic acid derivative	71.0	487	487, 457, 441, 439, 423, 395, 385, 355, 334, 302, 285, 235			✓		
	asiatic acid/madecassic acid derivative	71.2	473	473, 455, 453, 437, 409, 401, 371, 353, 319, 305, 265, 217, 135			✓		
109	asiatic acid/madecassic acid derivative	70.9	517	517, 455, 439, 421, 395, 379, 377, 311		✓	✓	✓	[8]
111	asiatic acid/madecassic acid derivative	72.4	487	487, 469, 437, 423, 405, 393, 377	✓	✓			[8]
112	asiatic acid/madecassic acid derivative	72.7	487	487, 469, 441, 423, 407, 393, 377, 361, 289, 239, 189	✓	✓	✓	✓	[8]
113	asiatic acid/madecassic acid derivative	73.8	487	487, 441, 423, 409, 407, 393, 353, 135		✓	✓	✓	[8]
114	asiatic acid/madecassic acid derivative	74.8	564	564, 505, 279, 261, 243, 146 109	✓	✓			[8]
	asiatic acid/madecassic acid derivative	75.0	483	483, 465, 455, 447, 439, 421, 405, 391, 353, 329, 283, 239			✓		
	asiatic acid/madecassic acid derivative	76.2	485	485, 467, 441, 437, 423, 393, 387, 377, 369, 339, 289			✓		
115	asiatic acid/madecassic acid derivative	78.6	633	633, 615, 589, 545, 529, 527, 495, 441, 409, 162	✓			✓	[8]
**Other Compounds/Undefined**									
3	undefined (dimer 183)	4.6	367	367, 331, 325, 283, 183, 139, 111, 95	✓	✓			
29	roseoside derivative (pentoside)	21.4	563	563, 517 [M − H] − 46, 385, 223, 205, 191, 179, 161, 153, 138, 113	✓		✓	✓	[23,50]
	emodin derivative (tentatively assigned)	22.4	415	461, 415, 269, 161				✓	
34	undefined	22.7	467	467, 458, 449, 436, 38, 299, 275, 229, 169	✓				
37	undefined	26.2	439	439, 393, 311, 261, 221, 191, 179, 161, 149, 131, 113	✓				[51]
66	undefined	45.6	523	523, 475, 432, 341, 329, 315, 314, 283, 149	✓	✓		✓	
68	undefined	47.1	517	517, 491, 487, 439, 341, 301, 291, 275, 259, 209, 195, 97	✓				
	undefined	47.1	523	523, 475, 432, 341, 329, 315, 314, 283, 149				✓	
	undefined	48.8	263	263, 245, 219, 204, 201, 186, 163, 161, 152, 119, 99			✓		
76	undefined	52.7	423	423, 279, 249, 205, 168, 139, 124	✓	✓	✓	✓	
79	undefined	55.2	523	523, 489, 313, 167, 149, 122	✓		✓		
82	undefined	56.8	501	501, 471, 443, 315, 290, 275, 259, 195, 97	✓				
	undefined	57.9	543	543, 767, 319, 301, 275, 169			✓		
87	undefined	59.6	503	503, 455, 443, 428, 382, 298, 270	✓		✓		
	oxo-dihydroxyoctadecenoic acid	63.6	327	327, 309, 291, 229, 211, 171			✓	✓	[47]
110	undefined	71.7	473	473, 455, 439, 422, 403, 367, 319, 263, 237		✓	✓	✓	
	9-oxo-octadecadienoic acid derivative	75.6	293	293, 231, 275, 265, 231, 211, 185, 183, 171, 149, 111			✓		[46]
	9-oxo-octadecadienoic acid derivative	75.8	293	293, 275, 265, 231, 224, 196, 195, 179, 177, 139, 113, 111			✓	✓	[46]
	unknown flavonoid	76.6	473	473, 453, 413, 369, 287, 271, 201			✓		
	fatty acid derivative	77.0	311	311, 291, 249, 233, 185, 181, 171, 155, 141, 127			✓		
116	undefined	78.9	663	663, 645, 619, 604, 587, 533, 505, 399, 331, 175	✓		✓	✓	

^a^ For peak assignment, see Figure 3. Compounds without numbers were not characterized in the oily extracts A and B (Figure 3). ^b^ Putative assignment. ^c^ HHDP: hexahydroxydiphenic acid. ^d^ Exact assignment to one compound not possible.

Upon comparison of the phenolic profiles (Table 1) of the previously defatted seeds with those of the cold-pressed oils, it becomes obvious that the latter were devoid of the numerous gallotannins (e.g., galloyl-HHDP-glucosides). In contrast, hydrolyzable tannins and further polar components were abundant in the methanolic seed extracts. The seed oils were characterized by the presence of flavonoids, phenolic acids, and triterpenoids. Furthermore, a lignan (pinoresinol derivative) and a sesquiterpene (abscisic acid derivative) were characterized. Previous studies of the roots and herbal parts of *G. urbanum* showed that the plant contains phenolic compounds (gallic acid, caffeic acid, chlorogenic acid, eugenol, flavonoids and tannins), vicianose and carotenoids [2,52,53]. Thus, more polar phenolic components of the seeds are partly or entirely discriminated upon oil recovery, presumably due to poor solubility in the fatty oil, and remain in the press cake. Ellagic acid derivatives were hardly present in the cold-pressed, light-green-colored seed oil of *G. rivale*. Furthermore, the cold-pressed oil of *G. urbanum* had a dark green color and was highly viscous. Since the extraction method can significantly influence the compound profile of an oil, a systematic comparison of different methods is of interest for future studies, including supercritical fluid extraction [54]. In contrast, the nonpolar bioactive triterpene derivatives of AA and MA were detected in the fatty oils of both species for the first time, which, in combination with the valuable fatty-acid profile, renders them promising sources for potential applications in the food, cosmetic, and pharmaceutical sectors. This favorable compound profile is attributed to the fact that triterpenoids may contribute to the bioactivity spectrum of fatty oils and that they may also have a structure-forming function similar to sterols, as postulated for olive oil [55,56,57].

### 2.3. Determination of Total Phenolic Contents in Seed Oils and Assessment of their Antioxidant Potential

**Figure 5:**
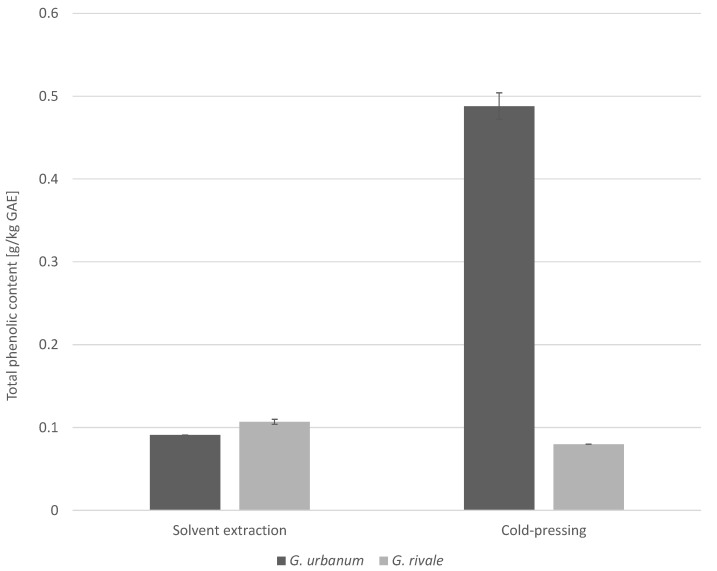
Determination of total phenolic contents of *G. urbanum and G. rivale* seed oils using the Folin–Ciocâlteu assay (*n* = 3).

Estimation of total phenolic contents using the Folin–Ciocâlteu (FC) assay revealed that *G. urbanum* and *G. rivale* seed oils recovered by solvent extraction showed nearly the same phenolic contents (Figure 5). These amounted to 0.091 ± 0 g gallic acid equivalents (GAE)/kg *G. urbanum* seed oil, whereas the oil of *G. rivale* seeds showed a phenolic content of 0.107 ± 0.003 g GAE/kg. Phenolic contents were also determined for the cold-pressed oils of both species revealing amounts of 0.488 ± 0.016 g GAE/kg (*G. urbanum*) and 0.080 ± 0 g GAE/kg (*G. rivale*), respectively. 

The difference in the phenolic contents of *G. urbanum* seed oil of 0.091 g/kg GAE (extraction) and 0.488 g/kg GAE (cold-pressing) is supposedly due to the difference of the extraction methods. Extraction with CH_2_Cl_2_ appeared to enhance the proportions of phenolics, such as HHDP derivatives, which are characterized by lower antioxidant capacity as determined by the FC assay. The difference between the two measured values of 0.107 g/kg GAE and 0.080 g/kg GAE of *G. rivale* seed oils are probably within the range of the natural variation of phenolic contents or result from the different extraction methods. 

It must be kept in mind that oil recovery by cold-pressing and solvent extraction may significantly differ with regard to the resulting compound profile and contents. For comparison, a study on extra virgin olive oil (EVOO) showed total phenolic contents to range from 0.138 to 0.278 g GAE/kg [58]. EVOOs are known for their abundance of phenolic compounds. Oils obtained by extraction and pressing may significantly differ in their metabolite profiles. Based on its physicochemical traits, CH_2_Cl_2_ selectively extracts nonpolar and moderately polar seed components. This may result in the discrimination of phenolics, thereby lowering the total phenolic contents in the extracted oil. Thus, for a true comparison, *Geum* seeds were also pressed to evaluate the phenolic contents of the respective seed oil. Phenolic compounds have a significant impact on the stability, sensory, and nutritional characteristics of plant-based products and may prevent deterioration through quenching of radical reactions responsible for lipid oxidation [59,60]. Such antioxidant effects may be assessed using the FC assay, which also indicates the antioxidant phenolic content of a sample. In particular, the cold-pressed seed oil of *G. urbanum* with its high phenolic content (0.488 g GAE/kg) may be promising from a nutritional viewpoint, as well as a high-quality oil for cosmetic purposes. Furthermore, previous studies reported total phenolic acid content (2,2-diphenyl-1-picrylhydrazyl radical scavenging activity, DPPH) of 0.768 ± 0.026 g GAE/kg (768.2 ± 25.9 mg GAE/L) of aqueous *G. urbanum* root extracts [61]. Other investigations of dried arial and underground parts of *G. rivale* reported phenolic contents nearly twice as high in the underground parts (17.48% GAE) compared to other plant materials tested (aerial parts: 7.83% GAE (*G. rivale*), 7.61% GAE (*G. urbanum*); underground parts: 7.89% GAE (*G. urbanum*)) with FC [62]. The high phenolic content and associated endogenous antioxidant activity of *G. urbanum* seed oil in the Folin–Ciocâlteu assay is presumably due to the presence of ellagic acid and its derivatives. Thus, future studies will have to focus on the natural variability of seed phenolic contents and profiles of *Geum* species from different origins.

## 3. Materials and Methods

### 3.1. Standards, Solvents and Reagents

A reference standard of madecassic acid was obtained from J&K Scientific GmbH (Pforzheim, Germany), and methyl γ-linolenate was obtained from Sigma-Aldrich (Taufkirchen, Germany). Methanol, methylene chloride, and acetonitrile were obtained from *Chemsolute* (Th. Geyer GmbH & Co. KG, Renningen, Germany). Formic acid was obtained from *Honeywell GmbH* (Seelze, Germany). For GC analyses, the methylation reagent trimethylsulfonium hydroxide (TMSH) was from *Fluka* (Buchs, Switzerland). A fatty-acid methyl ester (FAME) reference mixture C16–C24 for the identification of fatty acids was purchased from *Restek Corporation* (Bellefonte, PA, USA), and *tert*-butyl methyl ether (TBME) was obtained from *Merck* (Darmstadt, Germany). Folin–Ciocâlteu’s phenol reagent, gallic acid monohydrate, and sodium carbonate were from *Sigma-Aldrich* (Taufkirchen, Germany).

### 3.2. Plant Material

Seeds of *G. urbanum* L. and *G. rivale* L. were acquired from *Jelitto Perenial Seeds GmbH* (Schwarmstedt, Germany). Both species were identified by *Dr. R. Duque-Thüs*, and voucher specimens (voucher numbers: HOH-022758, HOH-022834 (*G. urbanum*); HOH-022759, HOH-022835 (*G. rivale*)) were deposited at the herbarium of the Institute of Botany at Hohenheim University (Stuttgart, Germany). In total, 520 g of *Geum* seeds were processed for all analyses performed in this work.

### 3.3. Extraction of Geum Seeds

Seeds of *G. urbanum* and *G. rivale* (20.0 g each) were separately immersed in 180 mL of CH_2_Cl_2_, and each batch was minced by *Ultra-turrax^®^* treatment (2 min; 17,000 rpm, IKA Werke GmbH & Co. KG, Staufen, Germany), under external ice cooling. After maceration over night at 4 °C, the seeds were filtered off over *Celite* by vacuum suction and extracted a second time in the same manner (overnight). Two oil fractions were recovered from the combined CH_2_Cl_2_ extracts by vacuum rotoevaporation of the solvent (oily extracts). Subsequently, the defatted seeds were extracted twice with MeOH (2 × 180 mL each) and filtered off; the extracts were combined and MeOH was removed in vacuo (rotary evaporation). 

In a second variant, oil was recovered using an oil press (Rommelsbacher OP 700 Emilio, ROMMELSBACHER ElektroHausgeräte GmbH, Germany). For this purpose, 100 g of *G. rivale* seeds and 100 g + 80 g of *G. urbanum* seeds were cold-pressed (T < 40 °C). The yielded oil crude extracts were centrifuged (10 min, 1000 rpm) to yield clear oil samples. (since *G. urbanum* seeds appeared to be very dry, another amount of 80 g together with the press residue of previously pressed seeds (100 g) was ground in the oil press for oil recovery). For phenolic compound analysis, aliquots of 2.5 g oil were dissolved in 5 mL of hexane and subsequently extracted two times with 5 mL of CH_3_OH–H_2_O (80:20, *v*/*v*) by 2 min vortex treatment and 5 min of centrifugation (4500 rpm). The methanol phases were removed by rotary evaporation, and the residues were dissolved in 1 mL MeOH/H_2_O (1/1; *v*/*v*) prior to HPLC-DAD/MS^n^ analyses. Each measurement was replicated three times (*n* = 3).

### 3.4. Methylation of Fatty Acids for GC/MS Analyses

Fatty-acid methyl esters (FAME) were prepared by column derivatization with trimethylsulfonium hydroxide (TMSH, 0.25 M in MeOH). Briefly, 10 mg of viscous oil sample (residue of CH_2_Cl_2_ extraction) was dissolved in 2000 μL of TBME. Aliquots of 10 μL of this test solution were mixed with 170 μL of TBME followed by 60 μL of TMSH [63]. Subsequently, the mixture was directly injected into the GC system (*n* =3).

### 3.5. Folin–Ciocâlteu Assay for Total Phenolic Content Determination and Assessment of Antioxidant Capacity

Total phenolic contents of plant extracts were determined employing the Folin–Ciocâlteu (FC) assay with gallic acid as standard [64]. For the analysis of oil samples, the method was adapted as follows: 2.5 g oil was diluted with 5 mL of *n*-hexane, and this solution was extracted two times with 6 mL of CH_3_OH–H_2_O (80:20, *v*/*v*) by 2 min vortex treatment and 5 min of centrifugation (4500 rpm). These extracts were analyzed in triplicate according to the following procedure: a portion of 1 mL of the extract was added to 0.25 mL of FC reagent (2 N) in a 10 mL volumetric flask. After 3 min at room temperature, 1.5 mL of Na_2_CO_3_ solution (20%, *w*/*v*) was added and mixed, and the volumetric flask was made up with purified water to the final volume. The samples were stored for 1 h at room temperature and centrifuged for 10 min at 12,000 rpm. Afterward, spectrometric analyses of the clear supernatant were performed at λ = 725 nm. Each measurement was replicated three times. The result, expressed in grams of gallic acid equivalents/kg, was calculated using a calibration curve established in a range of 0.05 to 0.17 mg/mL (concentrations: 0.05, 0.075, 0.1, 0.15, and 0.17 mg/mL). The standard curve of gallic acid was *y* = 4.5032*x* + 0.0574 with *R*^2^ = 0.9986 for the extracted seed oil samples (*n* = 3). The standard curve of gallic acid for the cold-pressed seed oil samples was *y* = 4.6544*x* + 0.0446 with *R*^2^ = 0.9992 (*n* = 4). 

### 3.6. GC/MS Analyses of Seed Extracts after Derivatization

GC/MS analyses were performed with a *PerkinElmer Clarus 500* gas chromatograph (PerkinElmer, Inc., Shelton, CT, USA) with split injection (split ratio 30:1, injection volume 1.0 μL), coupled to a single quadrupole mass detector. The column used was a *Zebron ZB-5MS* capillary column (60 m × 0.25 mm i.d. × 0.25 μm film thickness, 5% phenylpolysiloxane and 95% dimethylpolysiloxane coating; Phenomenex, Torrance, CA, USA). Carrier gas was helium at a flow rate of 1 mL/min. The injector used was a PSS (temperature-programmed split/splitless injector, temperature: 250 °C). The temperature program for the column oven was 100–320 °C with a linear gradient of 4 °C/min and a final hold time of 30 min. The mass spectrometer was run in electron ionization (EI) mode (70 eV). The software *Turbomass* (v.5.4.2, PerkinElmer Inc., Boston, MA, USA) was used for data acquisition and processing [65].

### 3.7. HPLC-(DAD)/ESI-MS^n^ Analyses of Phenolic Compounds

Liquid chromatographic analyses were carried out on an *Agilent 1200 HPLC* system (Agilent Technologies, Inc., Palo Alto, CA, USA), equipped with a binary pump, a micro vacuum degasser, an autosampler, a thermostatic column compartment, and a UV/Vis diode array detector. An *HCTultra* ion trap mass spectrometer (*Bruker Daltonik GmbH*, Bremen, Germany) with an ESI source operating in the negative ionization mode was coupled to the LC system, applying the following parameters: capillary voltage: +4000 V, dry gas (N_2_) flow rate: 9.00 L/min with a capillary temperature of 365 °C; nebulizer pressure: 50 psi. Full-scan mass spectra (mass range *m*/*z* 50–1300) of HPLC eluates were recorded during chromatographic separation yielding [M − H]^−^ ions. MS^n^ data were acquired in the auto MS/MS mode. The instruments were controlled by Agilent Chemstation and Esquire- Control software (V7.1). A *Kinetex*^®^ C18 reversed-phase column (2.6 μm particle size, 150 × 2.1 mm i.d., Phenomenex Ltd., Aschaffenburg, Germany) was used for chromatographic separation at 25 °C at a flow rate of 0.21 mL/min. The mobile phase consisted of HCOOH/H_2_O, 0.1/99.9 (*v*/*v*; eluent A) and MeCN (mobile phase B). The injection volume of each sample was 10 μL, and the gradient used was as follows: 0–8 min, 0–10% B; 8–20 min, 10% B; 20–51 min, 10–23% B; 51–70 min, 23–60% B; 70–80 min, 60–100% B; 80–85 min, 100% B; 85–90 min, 100–0% B; 90–100 min, 0% B [65].

## 4. Conclusions

This study reports, for the first time, the recovery of fatty oils from the seeds of *G. urbanum* and *G. rivale* and provides an in-depth analysis of the major constituents. In summary, an unsaturated oil with potentially biologically active phenolics may be recovered from the seeds. In particular, ellagic acid and HHDP derivatives were characterized in the solvent-extracted oils, which were also characterized in roots and herbal parts of these species in previous studies. In contrast, the latter compounds were only found at low concentrations in the cold-pressed oils. These oils are also particularly interesting due to the occurrence of triterpenoid derivatives of asiatic and madecassic acid, which have promising biological activities, such as anti-inflammatory, wound healing, and anticancer properties. In particular, the antioxidant, antibacterial, antifungal, and anti-inflammatory properties of the oils and the corresponding active principles should be investigated in more detail in future studies. Thus, the present study with its characterization of secondary metabolites provides a first step indicating the seed oils of *Geum* species as having a promising bioactivity profile.
